# Pancreaticoduodenectomy after distal gastrectomy: A case series

**DOI:** 10.1016/j.ijscr.2020.09.169

**Published:** 2020-10-01

**Authors:** Mizuki Fukuta, Atsushi Tomibayashi, Takao Tsuneki, Kohei Nishioka, Yuta Matsuo, Osamu Mori, Satoshi Fujiwara, Yasuhiro Yuasa

**Affiliations:** Department of Surgery, Tokushima Red Cross Hospital, Japan

**Keywords:** Pancreaticoduodenectomy, Gastrectomy, Pancreaticoduodenectomy reconstruction

## Abstract

**Introduction:**

Pancreatoduodenectomy (PD) after gastrectomy requires more attention during surgery than ordinary PD due to anatomical variation with different types of gastrointestinal anastomoses, the complication of anatomical dissection, and the extent of adhesions present. Herein, we describe the PD strategies we used in three patients who strategy for three patients who had previously undergone distal gastrectomy for gastric cancer treatment.

**Discussion:**

Generally, reconstruction following PD in patients who have undergone a prior Billroth I reconstruction is simple, as there is no previous afferent loop. By comparison, reconstruction after a Billroth II or R-Y reconstruction requires special consideration of the point of circulation and length of the remnant intestine used for pancreaticojejunostomy, choledochojejunostomy or gastrojejunostomy.

**Conclusion:**

There is no consensus regarding the best method for performing PD and reconstruction in patients with a prior history of gastrectomy. The appropriateness of using the existing afferent loop for reconstruction depends on whether the jejunum is of sufficient length for bile duct and pancreatic anastomosis. Regardless of the approach used, it is necessary to avoid excessive tension during reconstruction. Based on our experience and previous studies published in the English literature, we propose that reconstruction needs to be considered on a case-by-case basis.

## Highlights

• We describe the PD strategies we used in three patients who strategy for three patients who had previously undergone distal gastrectomy for gastric cancer treatment.
• PD after gastrectomy requires more attention during surgery than ordinary PD due to anatomical variation with different types of gastrointestinal anastomoses, the complication of anatomical dissection, and the extent of adhesions present, so the optimal method for reconstruction after PD should be considered on a case-by-case basis.

## 1 Introduction

According to the National Cancer Research Center Cancer Information Service in Japan, gastric cancer ranks first among all cancers in terms of incidence rate in men and third in women and is associated with a high rate of morbidity. Specifically, the morbidity rate of pancreatic cancer has been increasing among patients over the age of 75 years, in both men and women. In recent years, the outcomes of cancer treatment have improved, due to advances in diagnostic techniques and cancer treatments, leading to an increase in the incidence of pancreatoduodenectomy (PD) being performed as a treatment for a second primary cancer in older adults. As surgical treatment is the only curative treatment for these individuals, it is necessary to understand the resulting anatomical structure after gastrectomy; however, the best approach for pancreaticoduodenal resection following gastrectomy has yet to be defined [1]. In this case series report, we describe the PD procedures that we performed in three patients who had previously undergone gastrectomy for gastric cancer followed by reconstruction using a Billroth I or II anastomosis, or a Roux-en-Y (R-Y) gastrojejunostomy.

This work has been reported in line with the SCARE criteria [2].

## 2 Presentation of cases

### 2.1 Case 1

A 69-year-old man had undergone laparoscopic distal gastrectomy (LDG) followed by a Billroth II reconstruction in November 2015. Histological examination confirmed a pathological stage IV T4aN3bM1(Cy1) carcinoma, according to the TNM classification of the Union for International Cancer Control (7th edition). After surgery, the patient underwent chemotherapy, using the S-1 plus oxaliplatin regimen, for 3 months, followed by single-agent chemotherapy (S-1) for 1 year.

**Fig. 1:**
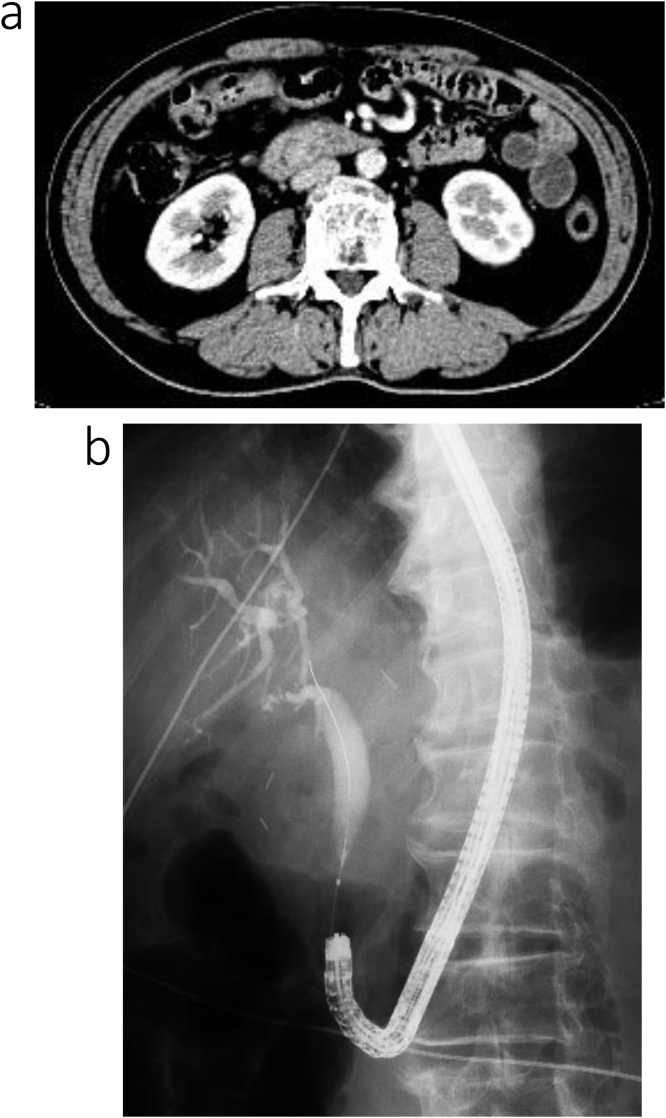
Contrast-enhanced computed tomography (CT) and endoscopic retrograde cholangiopancreatography (ERCP) of the patient in case 1. a. Contrast-enhanced CT images revealed dilatation of the intra- and extra hepatic bile duct due to a low-density 10-mm mass within the lower bile duct. b. ERCP revealed dilatation of the common bile duct and stenosis of the lower bile duct; cytodiagnosis was class V.

However, 27 months after gastrectomy, the patient’s carbohydrate antigen (CA) 19-9 serum level increased to 458 U/mL, although the patient did not present with any specific symptoms. Cytological examination of cells from the distal bile duct confirmed the diagnosis of lower bile duct cancer. The patient was re-admitted for treatment. Abdominal contrast-enhanced computed tomography (CT) and endoscopic retrograde cholangiopancreatography revealed dilation of the intra- and extra-hepatic bile ducts, secondary to the presence of a low-density, 10-mm, mass within the lower bile duct (Fig. 1a, b). Relevant findings of the serum blood analysis were as follows: hemoglobin, 13.1 g/dL; platelet count, 234,000/mL; total bilirubin, 1.4 mg/dL; aspartate aminotransferase, 84 U/L; and alanine aminotransferase, 81 U/L. Surgical treatment was selected as a curative method.

**Fig. 2:**
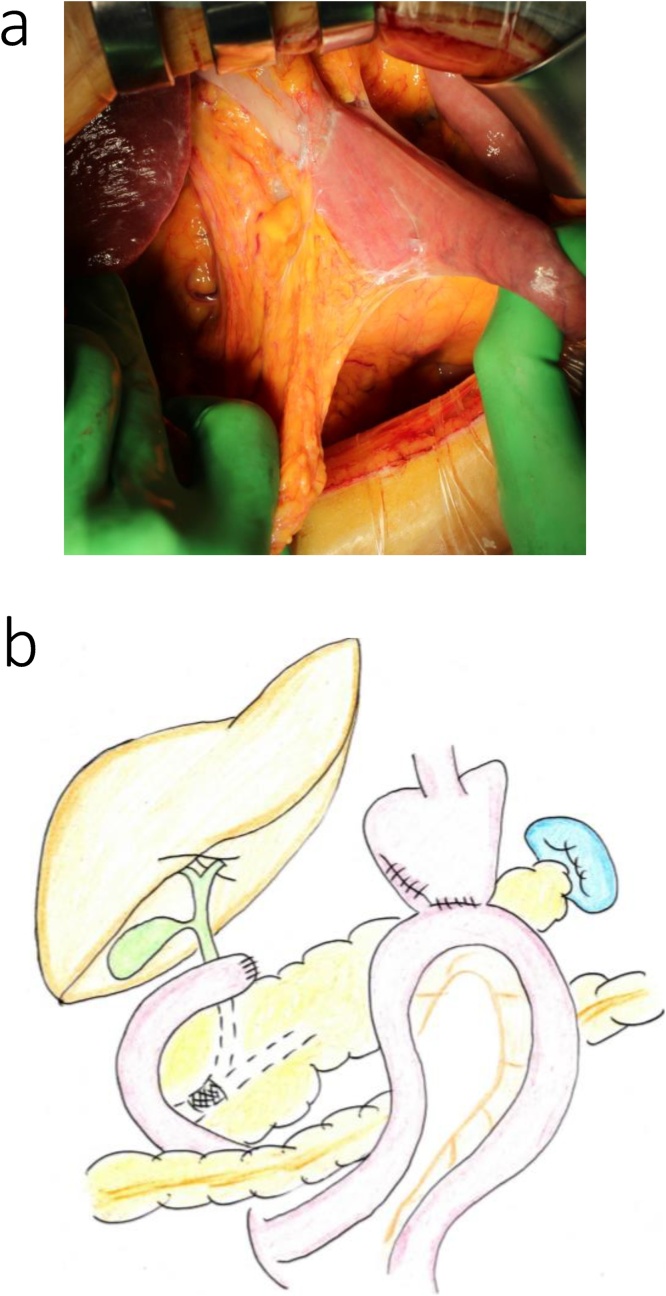
Intraoperative findings for the patient in case 1. a. Prior Billroth II reconstruction with adhesions at the gastrojejunal anastomosis. b. The excision range is demarcated by the red line, with the previous gastrojejunal anastomosis preserved.

**Fig. 3:**
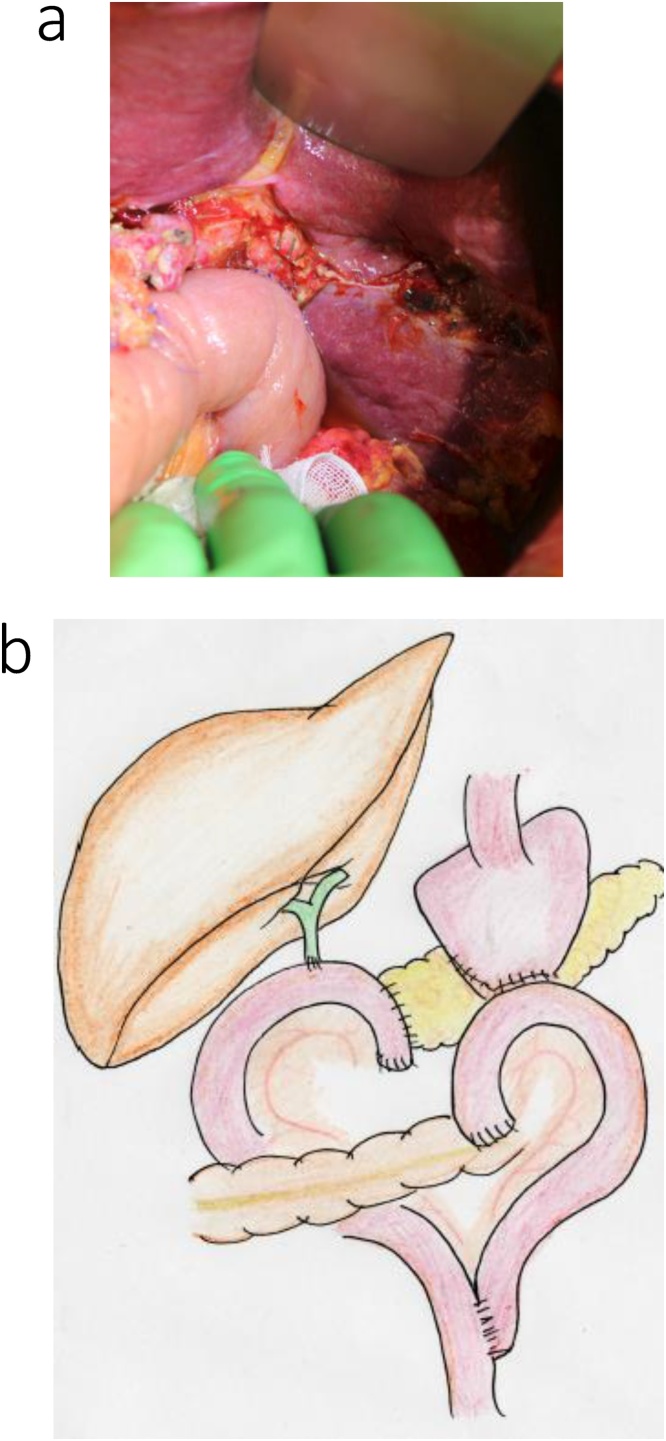
Intraoperative findings after reconstruction for the patient in case 1. In a and b, a new afferent loop was used for reconstruction.

During surgery, significant adhesions within the reticular cavity were observed immediately after making the upper abdominal midline incision, requiring exfoliation of the pancreatic head and mesocolon, from the right side, for anatomical orientation. In addition, marked fibrosis around the common hepatic artery, secondary to previous lymph node dissection, made identification of blood vessels difficult. Jejunal detachment was performed on the oral side of the gastrojejunal anastomosis site, preserving the previous gastrojejunal anastomosis site, and the pancreaticoduodenum was removed (Fig. 2a, b). The main pancreatic duct was 2-mm in diameter, and the pancreatic parenchyma was soft. We proceeded with a modified Child (subtotal stomach-preserving pancreatoduodenectomy [SSPPD]-IIA-1) reconstruction, with creation of a new afferent loop, but with preservation of the previous gastrojejunostomy (Fig. 3a, b). The operative time was 6 h and 49 min, with an intra-operative blood loss volume of 300 mL.

**Fig. 4:**
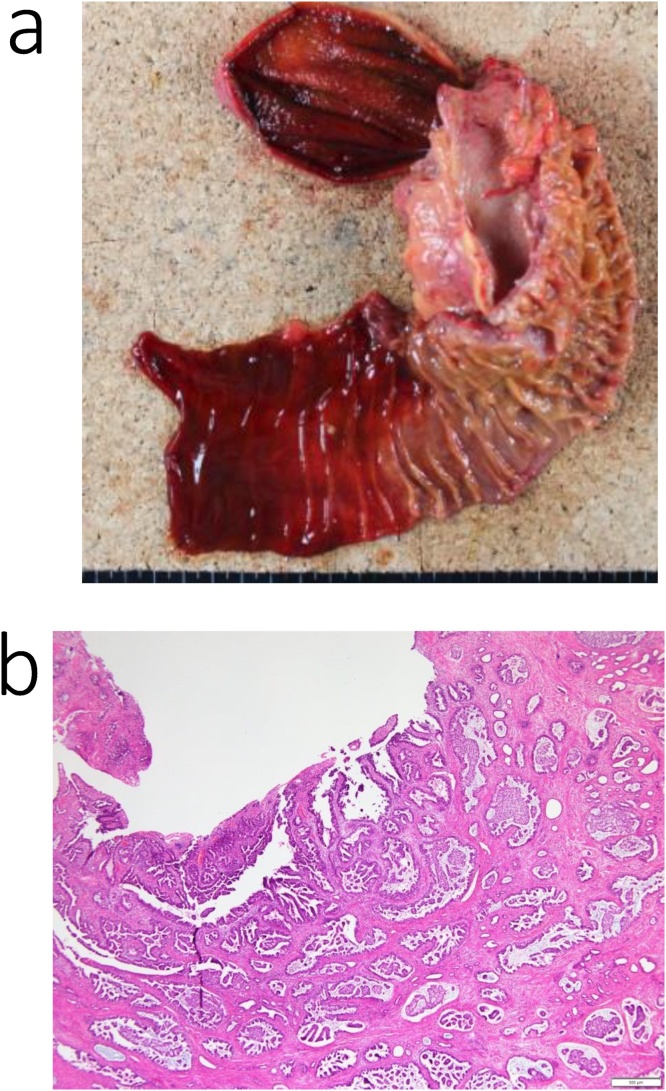
Pathological specimen from the patient in case 1. a. Excised specimen. b. The histological diagnosis of the pancreas head mass was metastatic adenocarcinoma (pap) of the stomach.

Histological examination of the mass in the pancreatic head confirmed a diagnosis of metastatic gastric adenocarcinoma (Fig. 4a, b). A pancreatic fistula (Grade B, based on the International Study Group of Postoperative Pancreatic Fistula) developed 7 days after surgery, which was treated conservatively. The patient was discharged on postoperative day 19.

### 2.2 Case 2

An 84-year-old woman had undergone LDG followed by a Billroth I reconstruction, in August 2014 for stage IA T1a(M)N0M0 gastric cancer, according to the TNM classification of the Union for International Cancer Control (7th edition). Adjuvant chemotherapy was not pursued after surgery, with no evidence of disease recurrence over a 4-year follow-up period.

**Fig. 5:**
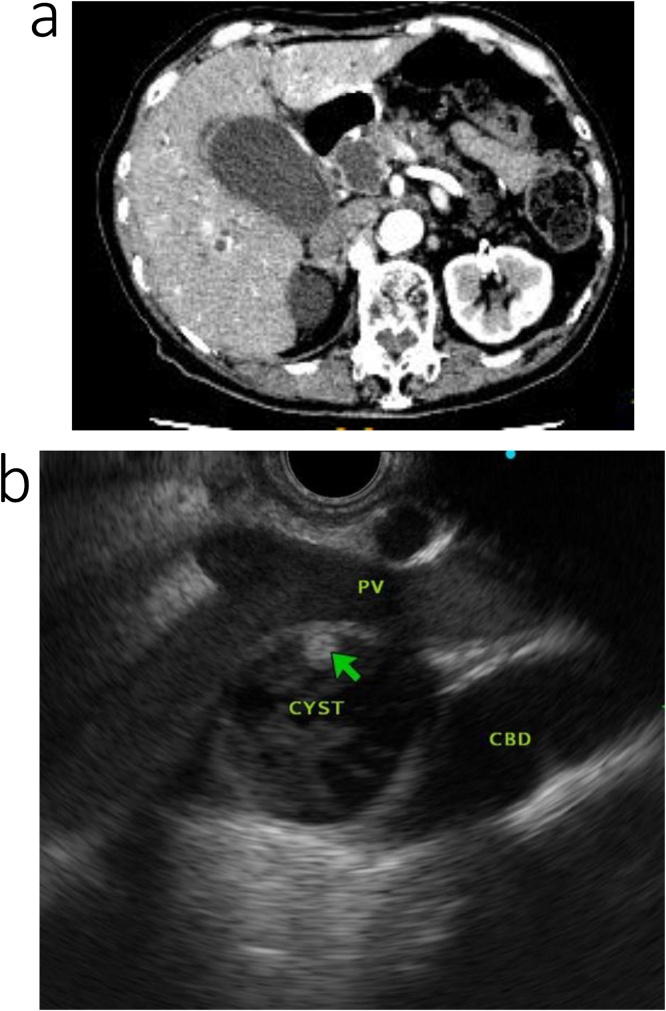
Contrast-enhanced computed tomography (CT) and endoscopic ultrasonography (EUS) for the patient in case 2. a. Contrast-enhanced CT images revealed dilation of the bile duct and of the common hepatic duct, with the contrast effect revealing a 26-mm cystic tumor in the head of the pancreas and mural nodules. b. A cystic tumor with mural nodules was found in the pancreatic head.

However, 48 months after gastrectomy, the patient was re-admitted for treatment of obstructive jaundice, and a diagnosis of intraductal papillary mucinous neoplasm (IPMN) was confirmed. Relevant findings of the serum blood analysis were as follows: hemoglobin, 9.3 g/dL; platelet count, 329,000/mL; total bilirubin, 6.6 mg/dL; aspartate aminotransferase, 105 U/L; and alanine aminotransferase, 104 U/L. The patient’s serum CEA and CA19-9 levels were elevated at 11.5 ng/mL and 299 U/L, respectively. Abdominal contrast-enhanced CT revealed dilation of the intra- and extrahepatic bile ducts and a 26-mm cystic tumor in the pancreatic head. On endoscopic ultrasound, a cystic tumor with mural nodules was also observed in the pancreatic head (Fig. 5a, b). Based on the IPMN diagnosis, we proceeded with surgical treatment.

**Fig. 6:**
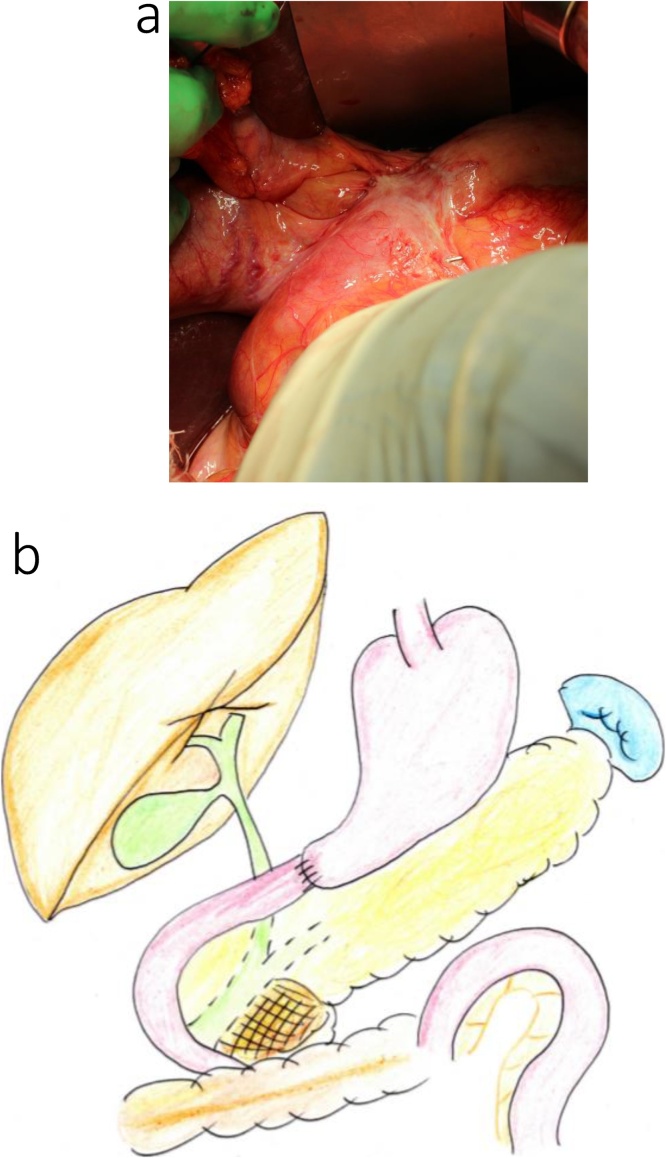
Intraoperative findings for the patient in case 2. a. Prior Billroth II reconstruction with adhesions at the gastrojejunal anastomosis. b. The excision range is demarcated by the red line, and the gastrojejunal anastomosis was resected.

**Fig. 7:**
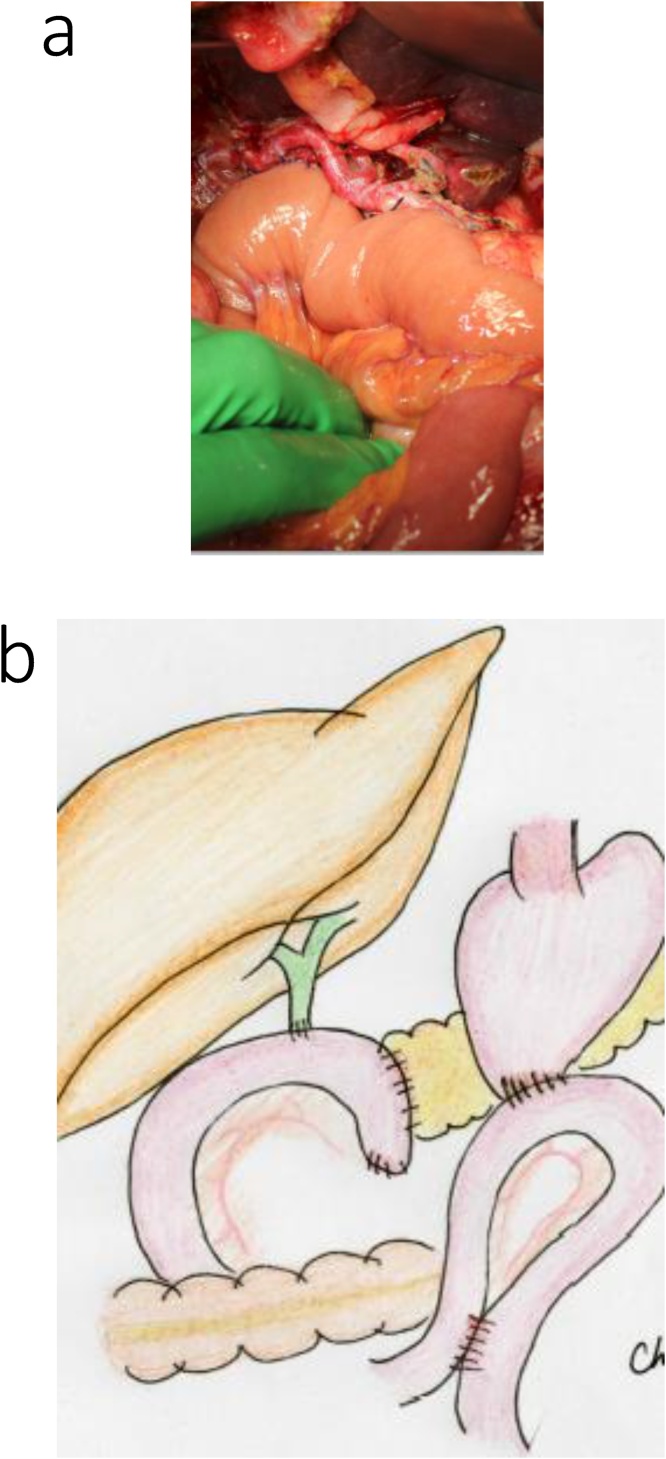
Intraoperative findings after reconstruction for the patient in case 2. In a and b, a new afferent loop was used for reconstruction.

A reverse L-incision was performed, with vascular detachment performed as that described in case 1. The previous gastroduodenal anastomosis site was resected, and the pancreatoduodenum was removed (Fig. 6a, b). The main pancreatic duct was 5-mm in diameter, and the pancreatic parenchyma was soft. A SSPPD-IIA-1 reconstruction was performed (Fig. 7a, b); the procedure was completed in 7 h and 38 m, with an intraoperative blood loss volume of 200 mL.

**Fig. 8:**
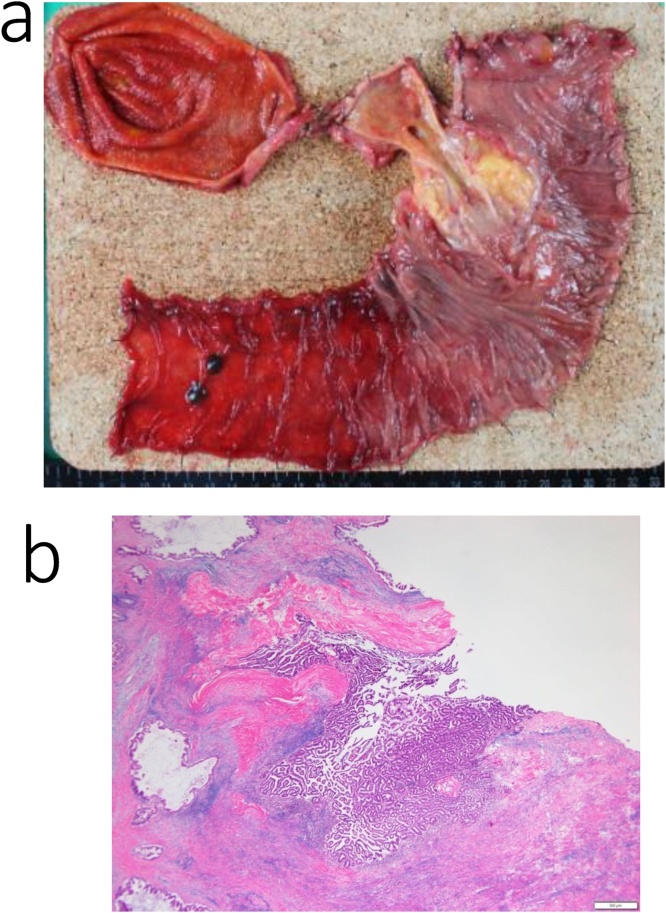
Pathological specimen from the patient in case 2. a. Excised specimen. b. The histological diagnosis of the pancreas head mass was an intraductal papillary mucinous carcinoma (IPMC).

Histological examination confirmed a diagnosis of intraductal papillary muscinous carcinoma of the pancreatic head (Fig. 8a, b). Chylorrhea developed on postoperative day 6, which was treated conservatively. The patient was discharged on postoperative day 20.

### 2.3 Case 3

A 64-year-old man had undergone distal gastrectomy followed by a R-Y reconstruction for gastric cancer (details unknown) in 2006, with no adjuvant chemotherapy treatment after surgery. The patient was followed for 6 years, with no evidence of disease recurrence.

**Fig. 9:**
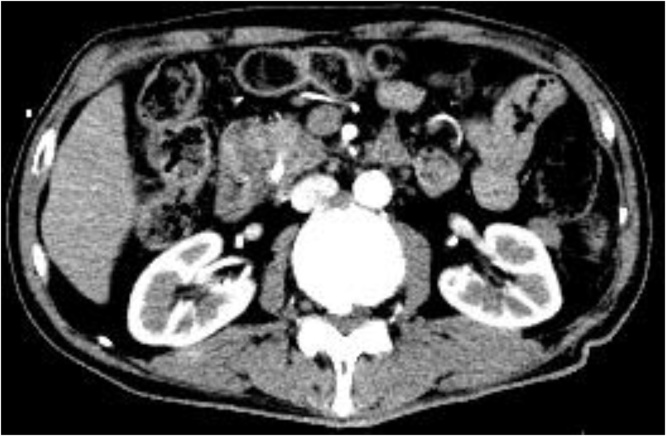
Contrast-enhanced computed tomography (CT) of the patient in case 3. Contrast-enhanced CT images revealed dilation of the intrahepatic bile duct due to a low-density tumor mass in the pancreatic head.

However, at 72 months after gastrectomy, the patient sought medical consultation at another hospital for obstructive jaundice and was subsequently transferred to our hospital for surgical treatment with a diagnosis of lower bile duct cancer. Relevant findings of the serum blood analysis were as follows: hemoglobin, 11.0 g/dL; platelet count, 268,000/mL; total bilirubin, 7.7 mg/dL; aspartate aminotransferase, 34 U/L; and alanine aminotransferase, 64 U/L. The patient’s serum levels of CEA, CA19-9, duke pancreatic monoclonal antigen type 2, and s-pancreas antigen-1 were elevated at 3.6 ng/mL, 48 U/L, 186 U/mL, and 21.9 U/mL, respectively. Contrast-enhanced CT revealed dilation of the intrahepatic bile duct secondary to a low-density tumor mass in the pancreatic head (Fig. 9). The patient was diagnosed with lower bile duct cancer, and we proceeded with surgical treatment.

**Fig. 10:**
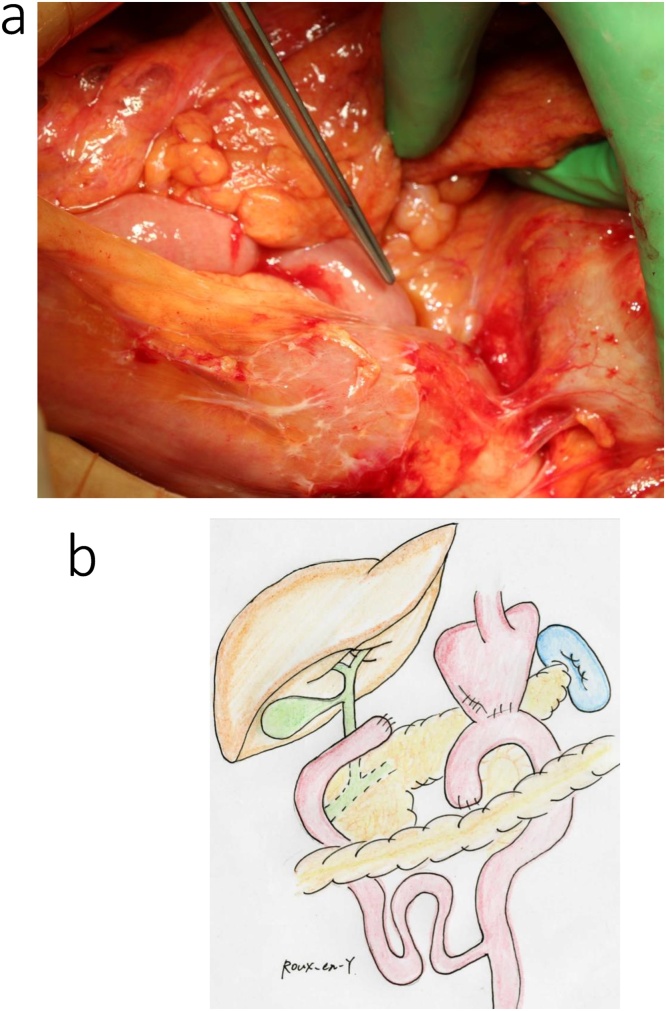
Intraoperative findings for the patient in case 3. a. A prior Roux-en-Y reconstruction with adhesions at the gastrojejunal anastomosis. b. The excision range is demarcated by the red line, and the afferent loop, from the Treitz ligament to the jejunal anastomosis, was relatively long, and the gastrojejunal anastomosis was preserved.

**Fig. 11:**
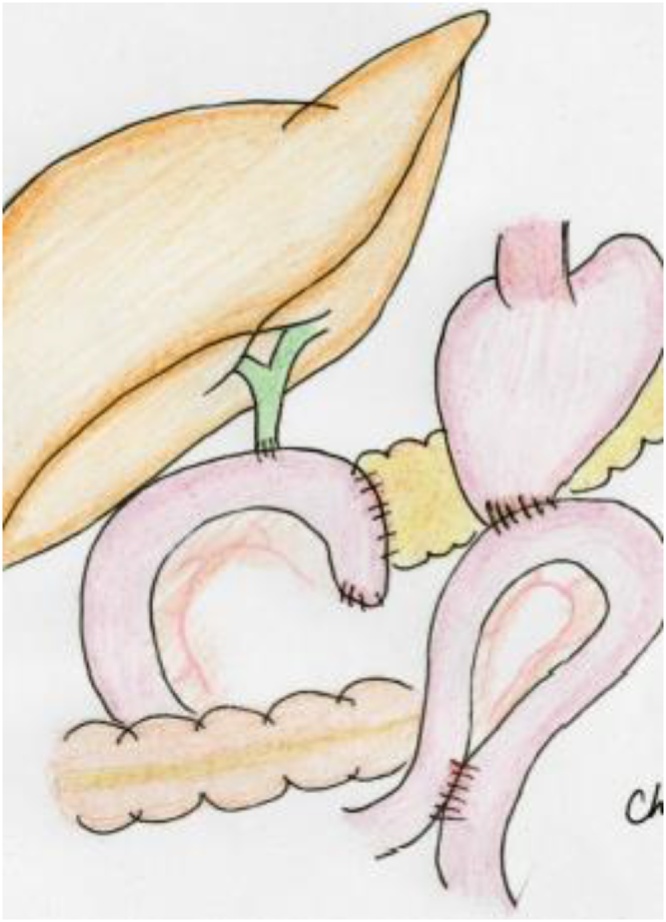
Reconstruction with the previous afferent loop in the patient in case 3. The existing loop was used for biliary jejunal anastomosis and reconstruction of the pancreatojejunal anastomosis.

The surgical procedure was performed via an upper abdominal midline incision. The afferent loop, from the Treitz ligament to the jejunal anastomosis, was relatively long (Fig. 10a, b), and was used for reconstruction of the choledochojejunostomy and the pancreaticojejunostomy (Fig. 11). The main pancreatic duct was 8-mm in diameter, and the pancreatic parenchyma was soft. The operative time was approximately 6 h and 41 min, with an intraoperative blood loss volume of 650 mL.

**Fig. 12:**
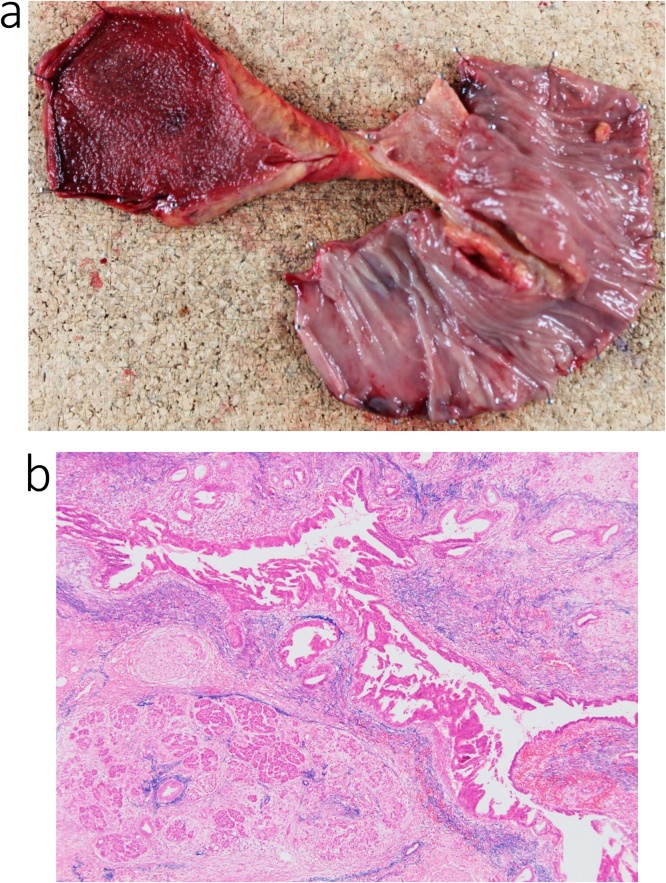
Pathological specimen from the patient in case 3. a. Excised specimen. b. The histological diagnosis of the pancreas head mass was pancreatic carcinoma.

The histological evaluation confirmed a diagnosis of the pancreatic head (Fig. 12a, b). The patient was discharged on postoperative day 20, without postoperative complications.

## 3 Discussion

As cancer treatments improve, the long-term survival of patients with gastric cancer is likely to rise annually [1]. As such, the likelihood of performing PD in patients who have previously undergone gastrectomy will also continue to rise [3]. PD procedures in these patients, however, are complicated due to multiple adhesions, secondary to scarring, from previous vasal exfoliation. Therefore, PD after gastrectomy is a challenging procedure, not only because of adhesions, but also because of variation in the anatomy of the remaining organs and the length of the remnant intestine [4]. In this report, we describe three cases of PD performed after afferent distal gastrectomy for gastric cancer with three different reconstruction procedures (Billroth I, Billroth II, or R-Y). We consider the following two points to be key decisions in these cases. The first is the decision regarding whether to preserve or resect the previous gastrointestinal anastomosis and the second is whether to preserve or resect the previous afferent loop in patients with a Billroth II or R-Y reconstruction.

Generally, reconstruction following PD in patients who have undergone a prior Billroth I reconstruction is simple, as there is no previous afferent loop. By comparison, reconstruction after a Billroth II or R-Y reconstruction requires special consideration of the point of circulation and length of the remnant intestine used for pancreaticojejunostomy, choledochojejunostomy or gastrojejunostomy [5]. We reviewed past cases of PD reconstruction after a prior Billroth II or R-Y reconstruction published in the English literature, using the keywords “pancreatoduodenectomy” and “gastrectomy” to identify the techniques used in these cases. Our search identified several relevant cases [1,[3], [4], [5], [6], [7], [8], [9], [10]], with a review of these cases indicating that a consistent opinion on the best approach has not yet been established.

Previous decisions regarding the preservation or resection of a previous gastrointestinal anastomosis in 51 patients [1,[3], [4], [5]], including our 3 patients, are summarized in Table 1. For patients who underwent a prior Billroth I reconstruction, the gastroduodenal anastomosis was resected in all patients. By comparison, for patients who underwent a prior Billroth II or R-Y reconstruction, the gastrojejunal anastomosis was preserved in almost all patients, except in cases in which adhesions were sufficiently strong such that there was a risk of damage at the time of exfoliation. In these cases, the gastrojejunal anastomosis was also resected. Therefore, the decision to resect or preserve a previous gastrointestinal anastomosis is generally made according to the previous reconstruction type.

**Table 1:** Summary of decisions to preserve or resect a prior gastrojejunostomy.

Reconstruction	Gastrointestinal anastomosis	
	Resected	Preserved
Billroth I (n = 13)	13(100%)	0
Billroth II (n = 24)	3(12.5%)	21(87.5%)
R-Y (n = 14) 0	14(100%)	

R-Y, Roux-en-Y reconstruction.

Regarding the preservation or resection of the previous afferent loop, 16 patients with clearly described postoperative complications were identified in the literature, in addition to our own 2 patients, with the decisions summarized in Table 2.

**Table 2:** Types of complications depending on whether the afferent loop was reconstructed.

Complication				
	PF	Bile leakage	Cholangitis	DGE
Billroth II (n = 4) + R-Y (n = 14)				
Afferent loop				
Resected (n = 12)	2(11%)			
Preserved (n = 6)			4(22%)	

PF, pancreatic fistula; DGE, delayed gastric emptying; R-Y, Roux-en-Y reconstruction.

In our case series, a long previous afferent loop was available in 1 patients, which required using the previous afferent loop for reconstruction, with no postoperative complications noted. Previous studies identified that the creation of a new afferent loop tended to reduce complications, especially cholangitis after PD, although using a long previous afferent loop can reduce the new anastomotic site and might shorten the operative time. Further, Lee et al. [1] reported that postoperative complications, could be reduced by reconstructing the gastrointestinal bile duct and pancreatic duct at the time of anastomosis, such that tension is not applied to the anastomosis. In other words, if the jejunum does not have sufficient length (>50 cm) to avoid excessive tension during reconstruction, the jejunum limbs will be shortened, resulting in the development of intractable cholangitis. Notably, no major complications were reported among 39 patients in whom the new afferent loop was used [1]. More recently, Kawamoto et al. [4] also confirmed the benefits of retaining a sufficiently long afferent loop to avoid complications after PD in a case series of 13 patients who underwent PD with reconstruction after prior gastrectomy.

This evidence should be considered when selecting the most appropriate reconstruction approach for a patient [[6], [7], [8], [9], [10]].

## 4 Conclusion

There is no consensus regarding the best method for reconstruction after PD in patients who have undergone prior gastrectomy with reconstruction for the treatment of gastric cancer. Generally, the afferent loop from the original reconstruction can be preserved if it is sufficiently long, which shortens the operative time. In the absence of strong evidence, the optimal method for reconstruction after PD should be considered on a case-by-case basis.

## Declaration of Competing Interest

The authors report no declarations of interest.

## Funding

There is no funding sources.

## Ethical approval

The ethical approval was given from our institution.

## Consent

Our patient has signed a consent form.

## Author’s contribution

Mizuki Fukuta: Operating Surgeon and writing the paper.

Atsusi Tomibayashi: Assistant Surgeon, Supervisor and writing the paper.

Takao Tsuneki: Writing the paper.

Kohei Nishioka: Writing the paper.

Yuta Matsuo: Writing the paper.

Osamu Mori: Writing the paper.

Satoshi Fujiwara: Writing the paper.

Yasuhiro Yuasa: Writing the paper.

## Guarantor

Mizuki Fukuta.

## Provenance and peer review

Not commissioned, externally peer-reviewed.
